# On the Value of Estimating Human Arm Stiffness during Virtual Teleoperation with Robotic Manipulators

**DOI:** 10.3389/fnins.2017.00528

**Published:** 2017-09-26

**Authors:** Jacopo Buzzi, Giancarlo Ferrigno, Joost M. Jansma, Elena De Momi

**Affiliations:** ^1^Department of Electronics, Information and Bioengineering, Politecnico of Milan, Milan, Italy; ^2^Mechanical Engineering Department, Delft University of Technology, >Delft, Netherlands

**Keywords:** human-robot interaction, surgical teleoperation, arm end-point impedance, musculoskeletal model, master devices comparison

## Abstract

Teleoperated robotic systems are widely spreading in multiple different fields, from hazardous environments exploration to surgery. In teleoperation, users directly manipulate a master device to achieve task execution at the slave robot side; this interaction is fundamental to guarantee both system stability and task execution performance. In this work, we propose a non-disruptive method to study the arm endpoint stiffness. We evaluate how users exploit the kinetic redundancy of the arm to achieve stability and precision during the execution of different tasks with different master devices. Four users were asked to perform two planar trajectories following virtual tasks using both a serial and a parallel link master device. Users' arm kinematics and muscular activation were acquired and combined with a user-specific musculoskeletal model to estimate the joint stiffness. Using the arm kinematic Jacobian, the arm end-point stiffness was derived. The proposed non-disruptive method is capable of estimating the arm endpoint stiffness during the execution of virtual teleoperated tasks. The obtained results are in accordance with the existing literature in human motor control and show, throughout the tested trajectory, a modulation of the arm endpoint stiffness that is affected by task characteristics and hand speed and acceleration.

## 1. Introduction

Teleoperated robotic systems are widely used in several long-short range application fields, from plant decommissioning (Cragg and Hu, 2003), environment exploration (Mitsou et al., 2006), hazardous material handling and surgery (Johnson and Somu, 2016). In order to control the remotely operated system, the user interacts with a master interface, a robot whose precise and accurate manipulation allows task execution at the slave side. The design of master interfaces is a fundamental aspect in teleoperation, indeed several studies focused on the master devices physical and control characteristics to achieve control transparency while assuring system stability (Fischer et al., 1990; Conti et al., 2014; Enayati et al., 2016). In teleoperation, stability of human-robot interaction (Colgate, 1994) is guaranteed by closing the control loop with the user sensory and motor systems and, in this context, both the robot and the user are considered as passive elements (Lee and Spong, 2006). Therefore, although the design of master devices architecture relies on the human user to achieve stability, the high variability that characterizes human control strategies are usually neglected. In fact, in human motor control, task stability is achieved by regulating the dynamic proprieties of the limbs through muscular activation. The arm impedance and its components (viscosity, inertia and stiffness) can be modified to adapt to different tasks and desired interactions by tuning muscle contractions and varying joint angular position,. As the most predominant component, the arm stiffness also directly depends on the joint angular velocity, reflex modulation and the presence of expected perturbations (Flash and Mussa-Ivaldi, 1990). Previous studies (Burdet et al., 2001) demonstrated the existence of control strategies employed to achieve stability through the regulation of the arm endpoint stiffness in terms of maximal value and orientation. Other studies showed the importance of stiffness regulation during complex tasks execution and motor learning (Osu et al., 2002; Gribble et al., 2003; Buzzi et al., 2017).

Up to now, the dynamic proprieties of the human arm have not been fully considered in the control and optimization design of the master interfaces (Hadavand et al., 2014; Qiu et al., 2014). Through robust and continuous estimation of arm stiffness, it would be possible to implement a master controller able to adapt and regulate the physical interaction between the robot and the human (Tsumugiwa et al., 2002; Ajoudani et al., 2012) in order to achieve better performance, higher resistance to external perturbations, and possibly reducing muscular fatigue (Wiker et al., 1989).

In order to estimate the human arm stiffness, several methods and devices have been proposed in literature: Flash et al. (Flash and Mussa-Ivaldi, 1990) as well as Gomi et al. (Gomi and Kawato, 1997) used planar robotized handles fitted with force sensors to record the interaction forces between the subjects hand and the robot when known displacements were applied. Force and displacement variations were used to compute hand stiffness in multiple directions. While these methods produce a measurement of the stiffness during postural maintenance, they cannot be applied to the study of stiffness during movement and task execution without interfering with arm kinematics. To overcome this limitation, microscopic displacements combined with a time frequency analysis were used to estimate the mechanical proprieties of the arm during a single reaching movement (Piovesan et al., 2013). Following an electromyography (EMG) based approach, recent works (Darainy et al., 2004; Shin et al., 2009; Ajoudani et al., 2015), estimated the arm endpoint stiffness using simplified planar musculoskeletal models and recorded surface muscle activations from couples of shoulder and arm antagonist muscles. Muscular models were used to estimate the force direction and arm of muscular units, while force intensities were obtained through calibration from the recorded EMG signals and the maximal voluntary contractions. Although the simplified musculoskeletal models showed the ability to estimate the arm endpoint stiffness during task execution and without applying perturbations to the user kinematics, stiffness computation was limited to specific planes and directions thus neglecting the effects of other couples of muscles, such as the wrist flexor/extensors.

In this work, we present a non-disruptive method for the computation of the arm endpoint stiffness based on a user specific 7 degrees of freedom (DoF) musculoskeletal model of the upper limb (Delp et al., 2007). The dynamic characteristics of the model as well as the activation dynamics of muscle units are used in conjunction with joint kinematics and EMG signals to obtain a continuous estimation of the arm stiffness (Pizzolato et al., 2015).

The aim of this work is to evaluate how different master devices and tasks influence the regulation of arm endpoint stiffness and its relation with task performance, hand speed and acceleration. We developed two planar tasks consisting in the position and orientation control of a virtual tool. The two task variations were intended to trigger the use of different biomechanical DoFs to demonstrate the influence of such constraints on the estimation of end-effector stiffness. We compared a parallel link (PL) and a serial link (SL) master device, to test how the differences between the two manipulators would elicit different levels of arm end-point stiffness during virtual teleoperation. During task performance, arm kinematics and EMG signals were acquired using an optical and a magnetic tracking systems).

Our primary hypothesis is that end-effector stiffness would be modulated according to both the mechanical features of the master device and the task characteristics. In particular, we expect the users to generate higher overall end-point stiffness when teleoperating with the serial link master device, characterized by lower structural stiffness and apparent mass. We also expect the users to increase arm stiffness while performing the second task; due to the increased complexity of simultaneously controlling position and orientation of the virtual tool. Our second hypothesis is that end-point stiffness would be modulated accordingly to curvature variations throughout the trajectory. More specifically, we believe that high curvature can be associated with higher complexity in executing the task, leading to higher values of arm end point stiffness. As third and final hypothesis, we expect a correlation between the hand speed and acceleration and arm end-point stiffness so that the users would generate the maximal levels of stiffness during slow and non accelerated movements.

## 2. Materials and methods

In order to analyze the stiffness regulation during virtual tele-operated tasks, we created a simple teleoperation scenario in which the users interacted with master devices to control virtual tools used to perform specific tasks.

### 2.1. Tasks design

Simple repeatable and cyclical planar virtual tasks were developed to challenge the users with different levels of complexity without requiring any surgical expertise.

**Figure 1:**
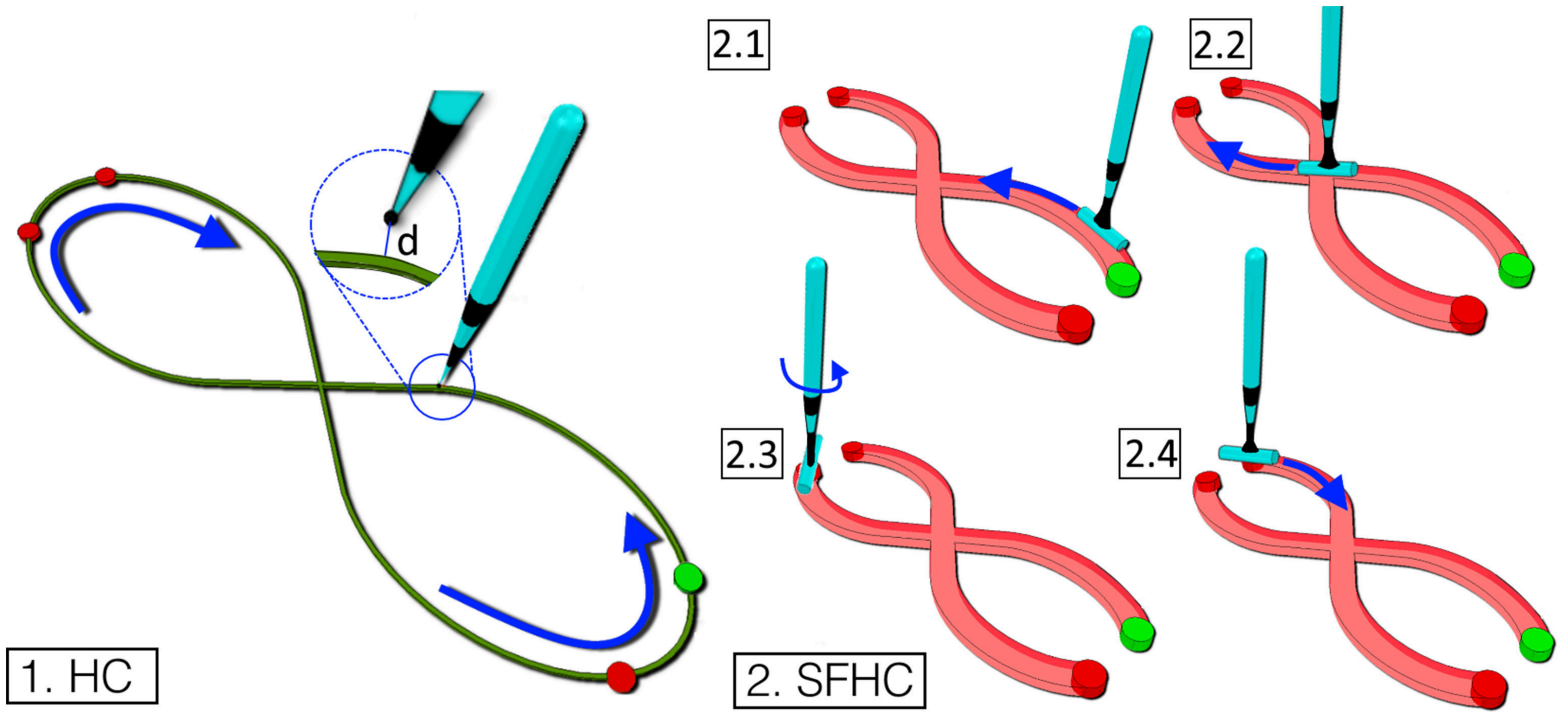
The figure represents the two trajectories designed. Task 1. A line following task shaped as an half cloverleaf (HC). The aim to to finely follow the trajectory starting from the green dot and moving anti-clockwise. Task 2. In the task, shaped as a thicker half cloverleaf (SFHC), the users have to orient the tool's cylindrical end-effector along the trajectory (2.1–2.4).

**Figure 2:**
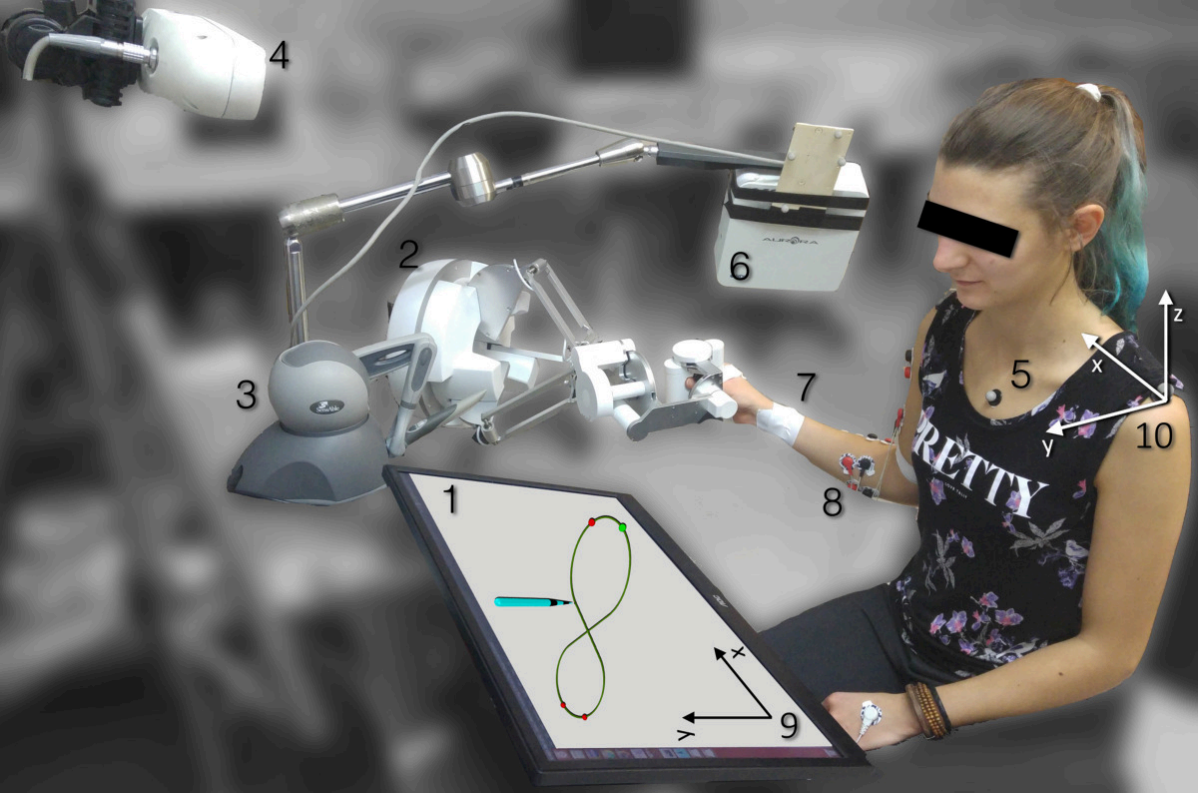
Experimental setup: the user performs the task looking at a monitor laid flat on a table (1) while teleoperating using either a parallel link haptic device PL (2) or a serial link haptic device SL (3) while the thorax and arm kinematic are acquired with an optical camera (4) and reflective markers (5) and with electromagnetic tracker (6) and markers (7). The EMG activation is also acquired with bipolar electrodes (8).The virtual reference frame (9) and the shoulder reference frame (10) are grossly aligned in the setup phase.

1. Half Cloverleaf (HC)
  Figure 1, 1 shows the first virtual trajectory which represents half the cloverleaf motion as presented by Levit-Binnun et al. (2006). A virtual stylus shaped tool was manipulated in the simulated environment to follow the trajectory, starting from the initial position (green dot in Figure 1, 1) and moving first counter-clockwise, then straight crossing the intersection in the middle, finally in a clock-wise direction to return to the starting position. In order to make the subject perform the task approximately in the x-y plane of a three-dimensional Cartesian reference frame, a visual cue was provided in the form of the path color which turned green when the tool-tip of the stylus was in the said plane (−1*mm* < *z* < 1*mm*), red otherwise.
2. Shape Fitted Half Cloverleaf (SFHC)
  The second task was designed to include wrist rotation as the subjects were asked to navigate a virtual cylindrical shaped tool tip along the path, as shown in Figure 1, 2. The trajectory that the users were requested to follow was identical to the one of the previous task, with the added complexity of following the trajectory silhouette using the tool end-effector. Starting from the green dot (Figure 1, 2), the users were asked to slide the tool end-effector over the shape fitted trajectory (see Figure 1, 2.1). In order to follow the first portion of the trajectory, the users needed to flex the wrist until the central intersection was reached (see Figure 1, 2.2) and then to extend it to reach the top left. At the trajectory upper end (red dot in Figure 1, 2.2), the users had to flex the wrist and abduct the shoulder to reposition the tool end-effector on the shape fitted trajectory (see Figure 1, 2.3). Similarly, the users had to reach the lower open end (see Figure 1, 2.4) to complete the task. As in the previous task, the visual feedback consisted in the change of the path color to green when the tool tip lied in the desired plane.

### 2.2. Experimental setup

#### 2.2.1. Master devices

The subjects were controlling the virtual tools position and orientation using two different master devices: a 7 DoFs parallel links haptic interface (PL) and a 6 DoFs serial links haptic interface (SL).

A Force Dimension Sigma7 (Force Dimension, Nyon, Switzerland) was used as PL (see Figure 2, 2). The master device, gravity compensated, is characterized by 6 DoFs plus a grip control, has a resolution of 0.0015 mm and 0.013° and an elliptical dome workspace with radiuses of approximately 190 × 130 mm. Thanks to its design, the translational and rotational degrees of freedom are completely decoupled, and the grasping unit has an apparent mass of 259 g (Tobergte and Helmer, 2013). The parallel-link structure also contributes to produce a system stiffness of approximately 14 *N*/*mm*.

**Figure 3:**
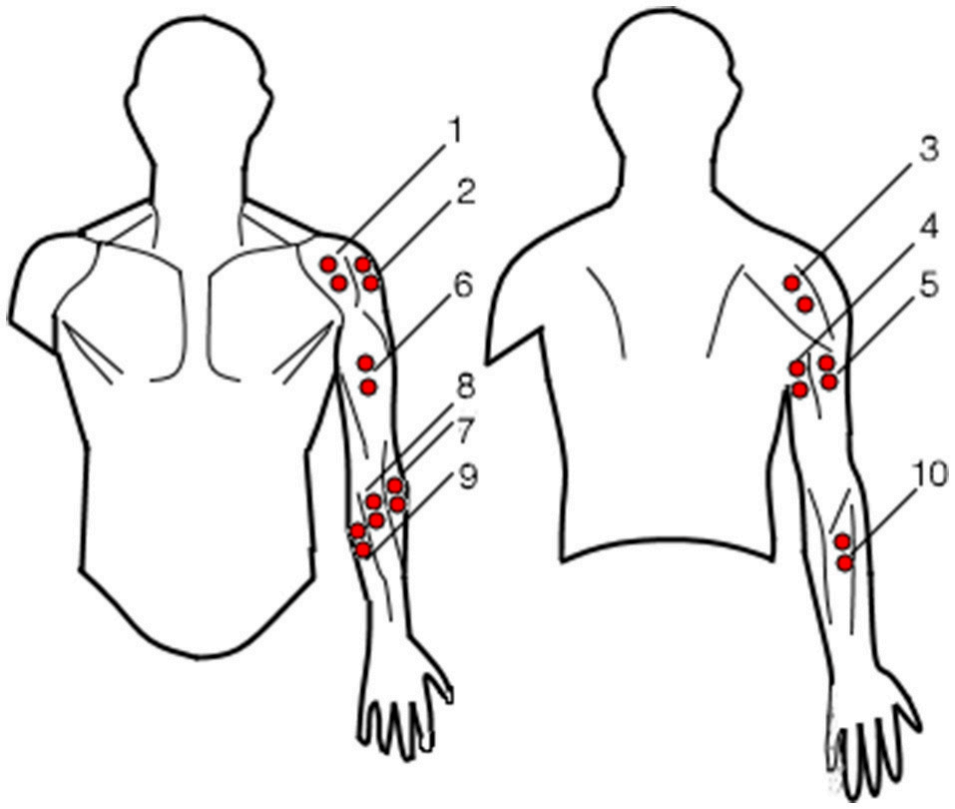
Surface electromyography electrodes placement: 1-2-3 for the anterior, lateral and posterior deltoids, 4-5 for the long and lateral trisceps brachii, 6 for the biceps, 7-8-9 for the brachioradialis, flexor carpi radialis and ulnaris respectively and 10 for extensor digitorum.

A Phantom Omni (3D Systems, South Carolina, USA) was used as SL (see Figure 2, 3). The device is characterized by a 0.055 mm resolution, a 160 × 120 × 70 mm workspace and its controlled through a stylus end-effector. Even though the device is not gravity compensated, which means that the tele-operator had to sustain the stylus and part of the links weight when manipulating it, it is characterized by an apparent mass at the tip of 45 g. Due to its design, the system stiffness components are not constant in the three axes, ranging from 1.02 *N*/*mm* to 2.31 *N*/*mm*.

Such differences in the master devices dynamic proprieties, both in terms of apparent mass of the end-effector and system stiffness, contribute to create a higher sense of stability while teleoperating with PL.

For both the SL and PL master devices custom impedance controllers were developed using the proprietary API. In both master devices, the users hand movements were downscaled with a factor of 2. The described tasks were designed to fit within the workspaces of both devices in order to avoid the necessity of using the devices clutching option. If used, the clutching would allow to decouple the virtual tool position from the master device end-effector position, allowing to reconfigure the arm when hitting the workspace limits. This option was excluded from the experiments since it would have caused significant kinematic variability during task execution.

#### 2.2.2. Acquisition framework

**Figure 4:**
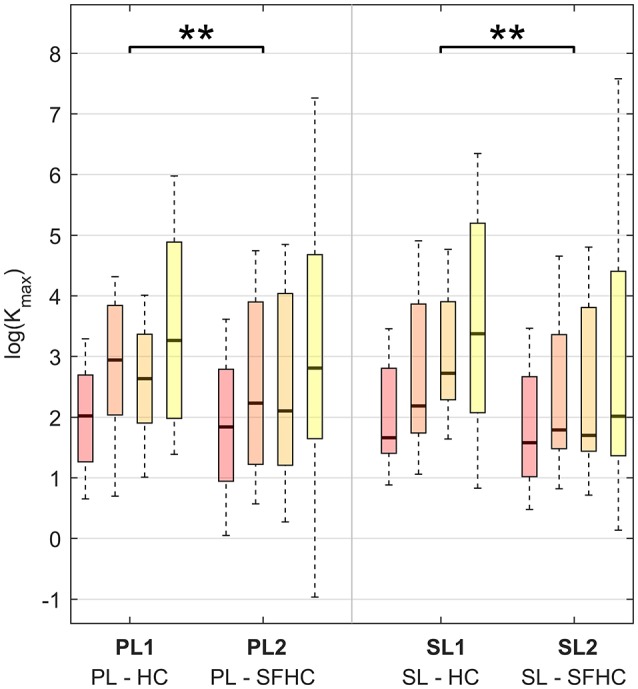
Maximal stiffness (*K*_*max*_) distribution in the four experiments for the four increasing levels of curvature. The results are presented using boxplot indicating median, first and third quartile, minimal and maximal values. Horizontal lines over the boxes indicate statistical difference while the number of stars indicate different levels of significance (^**^p < 0.01).

The users thorax and arm position and configuration were acquired using two localization devices calibrated to the same reference frame with a hand eye calibration approach (Horaud and Dornaika, 1995). The thorax position was acquired using an optical localization system (see Figure 2, 4) [Vicra, Northern Digital, Ontario, Canada, 20 Hz sampling rate, 0.25 mm position Root Mean Squared Error (RMSE)] using three passive retroreflective markers attached to the right and left acromions and next to the jugular notch.

The arm configuration was measured using an electromagnetic localization system (see Figure 2, 6) (Aurora, Northern Digital, Ontario, Canada, 30 Hz sampling rate, 0.48 mm and 0.3° position and orientation RMSE, dome shaped field with a radius of approximately 500 mm) and three 6-DoF 1.8 × 9 mm electromagnetic sensors (see Figure 2, 7) that were used to generate 6 corresponding virtual markers calibrated on the users recognizable bony landmark on elbow, wrist and hand.

EMG signals were recorded through a TMSi Porti device (Twente Medical Systems International, Oldenzaal, Nederland, 32 channel acquisition system, 2,048 Hz sampling rate) using 10 bi-polar electrodes (see Figure 2, 8). Three couples of electrodes were used to acquire the electromyographic signals from the anterior, lateral and posterior deltoid fiber bands (Figure 3, 1-2-3). Two couples of electrodes were used to acquire the long and lateral triceps brachii heads (Figure 3, 4-5) and a single couple was used to acquire the biceps muscle (Figure 3, 6). Four electrode couples were used on the forearm to measure the activation of the brachioradialis, flexor carpi ulnaris and radialis and extensor digitorum (Figure 3, 7-8-9-10). The mono-polar electrode used as reference was attached to the users' left hand. The muscular maximal voluntary contraction (MVC) was recorded right before the experiments: each subject was asked to perform a set of different isometric contractions against static resistance with different joint configurations (Lehman and McGill, 1999). The different movements were designed to elicit the activation of the muscles responsible for the same kinematic function: for instance, the biceps' MVC was recorded by asking the users to push the hand palm against a tabletop bottom while flexing the elbow; similarly, the forearm flexors' MVC were acquired during isometric wrist flexions against static objects. The contractions lasted for about 5 s and were followed by a second trial after 30 s, to avoid muscular fatigue. The mean of the two maximal absolute values registered during the two repeated movements was extracted from the conditioned signals for each muscle. The MVC signals were processed with the same procedure as the EMG signals recorded during the experiments.

Retroreflective and electromagnetic markers movements and EMG signals were acquired, recorded and synchronized using custom developed software based on the Robotic Operating System (ROS, http://www.ros.org/).

#### 2.2.3. Experimental protocol

We recruited 4 healthy subjects (2 female and 2 male, mean age 23 ± 1.5) who provided informed written consent, in accordance with the recommendations of Politecnico di Milano Ethical committee Board. All subjects gave written informed consent in accordance with the Declaration of Helsinki.

The subjects were sitting in a comfortable chair without arm rests, in front of a 2D monitor screen where the virtual tasks were displayed (Figure 2, 1). The chair position was adjusted in order to allow an easy interfacing between the subjects and the tasks. The monitor was laid as flat as possible on the table to be approximately parallel to the plane in which the tasks were performed, allowing for the most intuitive control, in which the users hand movement directly corresponded to the virtual tool movement.

Each user was asked to perform ten trials of every task while trying to maintain the hand movement on a constant plane, exploiting visual color feedback as explained in section 2.1. Subjects were instructed to perform HC and SFHC tasks finding a personal trade-off between precision and execution speed. Table 1 describes the four experiments performed: for each subject the experiment order was randomized so that users performed the 10 repetitions of a randomly selected condition before moving on the next randomly selected one.

**Table 1:** Experiments list and description.

**Experiment acronym**	**Master device**	**Task**	**Number of trials**
PL1	PL	HC	10
PL2	PL	SFHC	10
SL1	SL	HC	10
SL2	SL	SFHC	10

### 2.3. Stiffness computation

The kinematic and dynamic characteristics of the user arm movements, as well as their muscular activations were obtained and elaborated to compute the arm end-point stiffness during task execution.

#### 2.3.1. Kinematic analysis

The arm and thorax spatial configuration were reconstructed using the experimental markers data and the musculoskeletal model implemented in OpenSim (Delp et al., 2007). The model, whose capabilities in predicting the arm dynamics were previously assessed (Saul et al., 2015), is derived from Holzbaur's model (Holzbaur et al., 2005) and composed by seven DoFs activated by 32 muscle compartments. The model was scaled to fit the anthropometric characteristics of each subject and its virtual markers were displaced to the average real markers position acquired during a static pose.

The markers position data were filtered by applying an IIR second order Butterworth filter twice, reversing the time in the second filtering in order to cancel the non linear phase shift. Filter cutoff frequency was set to 4 Hz (-6 dB) and the filtered data were used as input for the inverse kinematics. The inverse kinematic algorithm solves the minimization problem in Equation (1) for each set of marker coordinates.

(1) $$\min_{q}[\sum_{k\in N=number\;of\;markers}w_{k}\|x_{k}^{exp}-x_{k}(q){\|}^{2}]$$

Where **q** is the vector of joint angles, ${\text{x}}_{k}^{exp}$ is the experimental 3D position of the *k*^*th*^ marker, acquired using the optical or the magnetic tracking device, **x**_*k*_(**q**) is the position of the corresponding virtual marker on the model that depends on the vector of joint angles **q**, and *w*_*k*_ is the *k*^*th*^ marker weight thorough which is possible to account for the uncertainty related to each marker position detection. For each set of marker coordinates, the algorithm finds the best joint vector **q** that minimizes the error between the experimental marker position vector **x**^*exp*^ and the virtual markers position vector *x*(**q**), which is a function of the fixed model geometry and joint vector, **q**.

In order to define the correct weighting vector **w** = [*w*_1_, …, *w*_*k*_, …, *w*_*N*_] with *N* = number of markers, we analyzed the markers positions during a static recording. The ratio between the standard deviations from optical markers and electromagnetic markers is comparable with the ratio between the RMSE characteristics of each device. We therefore weighted the thorax optical markers with a coefficient of 1, while the arm, forearm and hand electromagnetic markers with a coefficient of 0.52 (obtained from the ratio between the nominal RMSE for the optical tracker, 0.25 mm and the electromagnetic tracking device RMSE, 0.48 mm).

#### 2.3.2. Dynamic analysis

The obtained joint angles were filtered with an IIR second order Butterworth filter (cutoff frequency 4 Hz) twice, compensating the nonlinear phase shift. The filtered data were used as input for the inverse dynamic reconstruction, along with the model dynamic characteristics (masses and inertial proprieties of each model segment). Equation (2) shows the dynamic equation when no external forces are applied to the arm; **τ** is the unknown (7 × 1) vector of joint torques, **M** is the system mass matrix, **C** is the vector of Coriolis and centrifugal forces, **G** is the vector of gravitational forces, while ***q***, $\dot{\text{q}}$, $\ddot{\text{q}}$ are the vectors of joint position, velocities and accelerations, respectively.

(2) $$\tau =M(q)\overset{..}{q}-C(q,\dot{q})-G(q)$$

#### 2.3.3. EMG-informed torque estimation

A refined estimation of the joint torques was obtained using the Calibrated EMG-informed Neuro-Musculoskeletal (CEINMS) modeling toolbox in which the musculotendon unit (MTU) force arms obtained with the inverse kinematics are used with the joint torques estimated by the inverse dynamics and the recorded EMG signals.

The tool firstly calibrates the experimental muscle excitations derived from the surface EMG acquisition with the muscle activation patters, obtained from the inverse dynamic algorithm (Lloyd and Besier, 2003; Sartori et al., 2012). The resultant calibrated model is then used to predict MTU forces, joint moments, and muscle activations solving a set of differential equations that relates the muscle excitation with the electrical activity recorded (Shao et al., 2009). To be used as an input to the EMG-informed inverse dynamic algorithm, the EMG data was previously high pass filtered at 30 Hz, full wave rectified and filtered with a zero-lag second order Butterworth filter (6 Hz cutoff frequency) (Lloyd and Besier, 2003). The experimental muscle excitations were normalized using each users maximal voluntary contractions (MVC).

#### 2.3.4. Stiffness computation

For each model DoF, the joint stiffness (7 × 7) matrix (**K**_**j**_) was computed using Equation (3), where **τ**(*i*) is the joint torque vector that is associated with the **q**(*i*) arm configuration at the *i*^*th*^ time instant (McIntyre et al., 1996).

(3) $$K_{j}(i)=\frac{d\tau (i)}{dq(i)}$$

In order to obtain the Cartesian representation of the arm stiffness, the arm end-point Cartesian stiffness (**K**_**e**_) has been obtained using the arm kinematic Jacobian **J** (7 × 6), as follows:

(4) $$K_{e}(i)=J^{T^{-1}}(i)K_{j}(i)J^{-1}(i)$$

Through the Singular Value Decomposition (SVD), the left singular vectors and non-zero singular values of **K**_**e**_ were obtained to draw the end-point stiffness ellipsoid orientation and dimensions that are used as a way of visually represent the stiffness (McIntyre et al., 1996). The maximal singular value of **K**_**e**_ is the maximal stiffness value (*K*_*max*_(*i*)) in the *i*^*th*^ time instant.

### 2.4. Metrics and statistical analysis

The following performance-related parameters were evaluated:

- 2D distance from the trajectory at the *ith* time frame, *d*_*i*_: the distance is computed from the tool tip to the closest point on the trajectory center on the task plane (see Figure 1); it is calculated as shown in Equation (5).
  (5) $$d(i)=\sqrt[{2}]{{\left(x_{target}(i)-x_{tool}(i)\right)}^{2}+{\left(y_{target}(i)-y_{tool}(i)\right)}^{2}}$$
  Where (*x, y*)_*target*_ are the tool tip's 2D coordinates and (*x, y*)_*tool*_ are the 2D coordinates of the closest point on the trajectory. The z-component of the displacement from the trajectory was excluded from the distance metric computation due to the significant differences that the color visual feedback could introduce with respect to the other two dimensions.
- Maximal stiffness ***K***_*max*_: the end-point stiffness ellipsoid main axis was computed in 29 (for HC) and 23 (for SFHC) equally spaced points along the task trajectory (for SFHC, the points corresponding to the open ends were excluded from the analysis). For each task trial and for each point selected, the three closest virtual tool positions were searched and the corresponding maximal stiffness (***K***_*max*_(*j*), ***K***_*max*_(*j* + 1) and ***K***_*max*_(*j* − 1)) were averaged to obtain the mean maximal stiffness estimation as in Equation (6)
  (6) $${\bar{K}}_{max}=\frac{1}{3}(K_{max}(j-1)+K_{max}(j)+K_{max}(j+1))$$
  With *j* = 1…*M* − 1 number of points along the trajectory.
- Curvature *C*: In both tasks, the trajectory was created to fit the same Bernulli's Lemniscate function. The generic function of a Bernulli Lemniscate curve in a planar x-y plane with the main axis oriented along the x axis is
  (7) $${\left(x^{2}+y^{2}\right)}^{2}=2a^{2}(x^{2}-y^{2})$$
  Where *a* is the parameter that defines the position of the curve foci. The task trajectory curvature (*C*) can be computed in polar coordinates using:
  (8) $$C(t)=\frac{3*\sqrt{2}*cos(t)}{\sqrt{3-cos(2t)}}$$
  with *t* that spans from 0 to 2π. For each 3D point of the trajectory selected for the stiffness analysis, the corresponding curvature was computed.
- Hand speed and acceleration: the position and orientation of the hand in the shoulder reference frame (see Figure 2, 10) was computed. The speed was obtained performing numerical differentiation on the *x-y-z* coordinates, smoothing the signal with a second order Butterworth filter with cutoff frequency of 4 Hz twice forward and backward. Acceleration was computed and filtered from hand speed following the same procedure previously described.

For all the metrics, the statistical distribution normality was tested for each user in the four experiments PL1, PL2, SL1, SL2 (see Table 1) using a one-sample Kolmogorov-Smirnov test with 1% significance level. The arm stiffness data resulted to be non-normal, therefore, using the natural logarithm function (as in Wilson and Worcester, 1945), the data was normalized: the same Kolmogorov-Smirnov test was performed for each user, repetition and experiment, identifying as normal the 97% of the distributions. The distance metric, as well as the hand speed and acceleration had a normal distribution (Kolmogorov-Smirnov α = 0.01).

#### 2.4.1. Analysis performed and hypothesis tested

All the inferential statistic analysis that will be presented were conducted with the Statistics and Machine Learning Toolbox for Matlab 2016b (Mathworks, Natick, Massachusetts, US).

1. Maximal stiffness through the trajectory - The stiffness maximal values *K*_*max*_ were first of all studied as a function of the task type and controller. The distributions of the normalized stiffness data were compared between users and repetition, since no significant differences were found, the users data were grouped together for each experiment
  Hypothesis 1 & 2: To analyze whether the task and master device types, as well as level of trajectory curvature affected the maximal values of stiffness, the data from the 29 (HC) and 23 (SFHC) points on the trajectory for each experiment were grouped. The mean values of maximal stiffness for each region of curvature and repetition were extracted and a three-way ANOVA was performed on the log-normalized data (task, master device type and curvature region as fixed factors, user number as random-effect factor). To further test the effects of the different factors on the end-point stiffness, the same ANOVA model was also applied to the stiffness variability in the various regions and repetitions. To test the presence of possible correlations between stiffness and curvature the Pearson rank test (α = 0.05) was adopted.
2. Stiffness against hand speed and acceleration - Using two separate two-ways ANOVAs (task and master device type as fixed factors, user number as random-effect factor) the hand speed and acceleration distributions in the four experiments were analyzed. Hypothesis 3: we tested the hypothesis that a relation between hand speed and acceleration with respect to maximal stiffness could exist. For each of the ten trajectory repetitions, 4 levels of hand speed and acceleration were obtained in the four experiments and for the 4 users. Two separated three-way ANOVAs were performed on the corresponding values of stiffness (task, master device type and level of speed/acceleration as fixed factors and user number as random effect factor).
3. Distance metric - The difference between different trials of the same experiment for each user was tested using a one-way ANOVA test (α = 0.05). Since no statistical difference emerged, the mean values of distance throughout each repetition and user were extracted. The four experiments' performance distributions were then modeled with a two-way ANOVA statistical model, where task and master device type were considered as fixed factors, while user number was considered as random effect factor.

## 3. Results

### 3.1. Maximal stiffness through the trajectory

**Figure 5:**
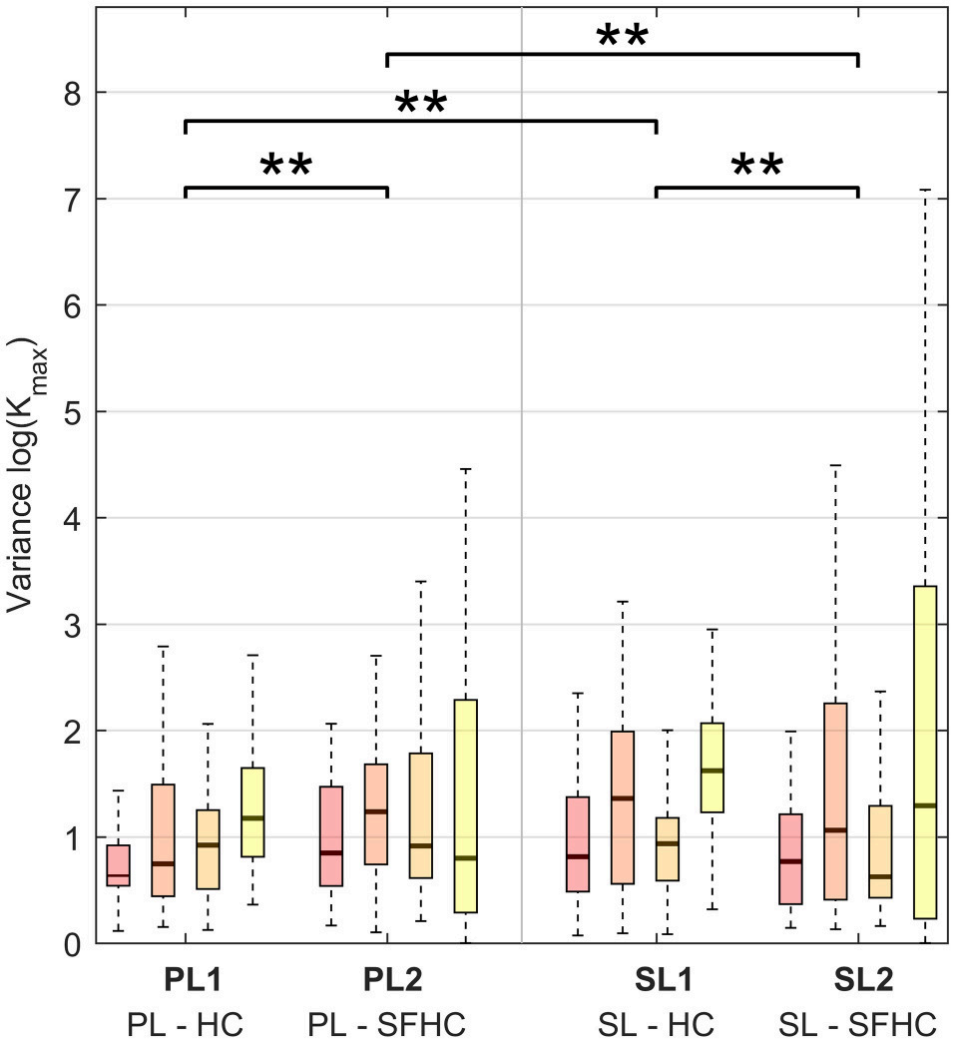
Variance of maximal stiffness (*K*_*max*_) in the four experiments for the four increasing levels of curvature. Horizontal lines over the boxes indicate statistical difference while the number of stars indicate different levels of significance (^**^p < 0.01).

Figure 4 shows the results obtained when the mean stiffness values through the trajectory among users and trials for each experiment are grouped together. The stiffness values below a threshold of 0.1 *N*/*m* were removed from the analysis: the threshold was obtained from previous arm end-point stiffness estimations (Gomi and Kawato, 1997; Tsumugiwa et al., 2002) and the percentage of values discarded was less than 1% of the total dataset. The results of the three-way ANOVA analysis showed no significant interaction between the factors, and a statistical difference between tasks (HC and SFHC) [*F*_(1, 624)_ = 10.06, *p* < 0.005] while no significant difference was found between the master devices [*F*_(1, 624)_ = 0.14, *p* = 0.70]. Overall, users elicited significantly higher arm stiffness when performing the HC task with respect to SFHC. Figure 5 shows the end-point stiffness variability in the four experiments. The three-way ANOVA test showed no significant interactions among factors and statistical difference between both tasks [*F*_(1, 624)_ = 7.75, *p* < 0.01] and master devices [*F*_(1, 624)_ = 7.64, *p* < 0.01]. Interestingly while the stiffness variance increases in the SFHC task when teleoperating with PL, when teleoperating with the SL master device, instead, users decreased the arm stiffness variance when performing SFHC with respect to HC. Overall, users explored the highest range of end-point stiffness in HC when teleoperating with SL and showed the lowest variance with the same master device in the SFHC task.

**Figure 6:**
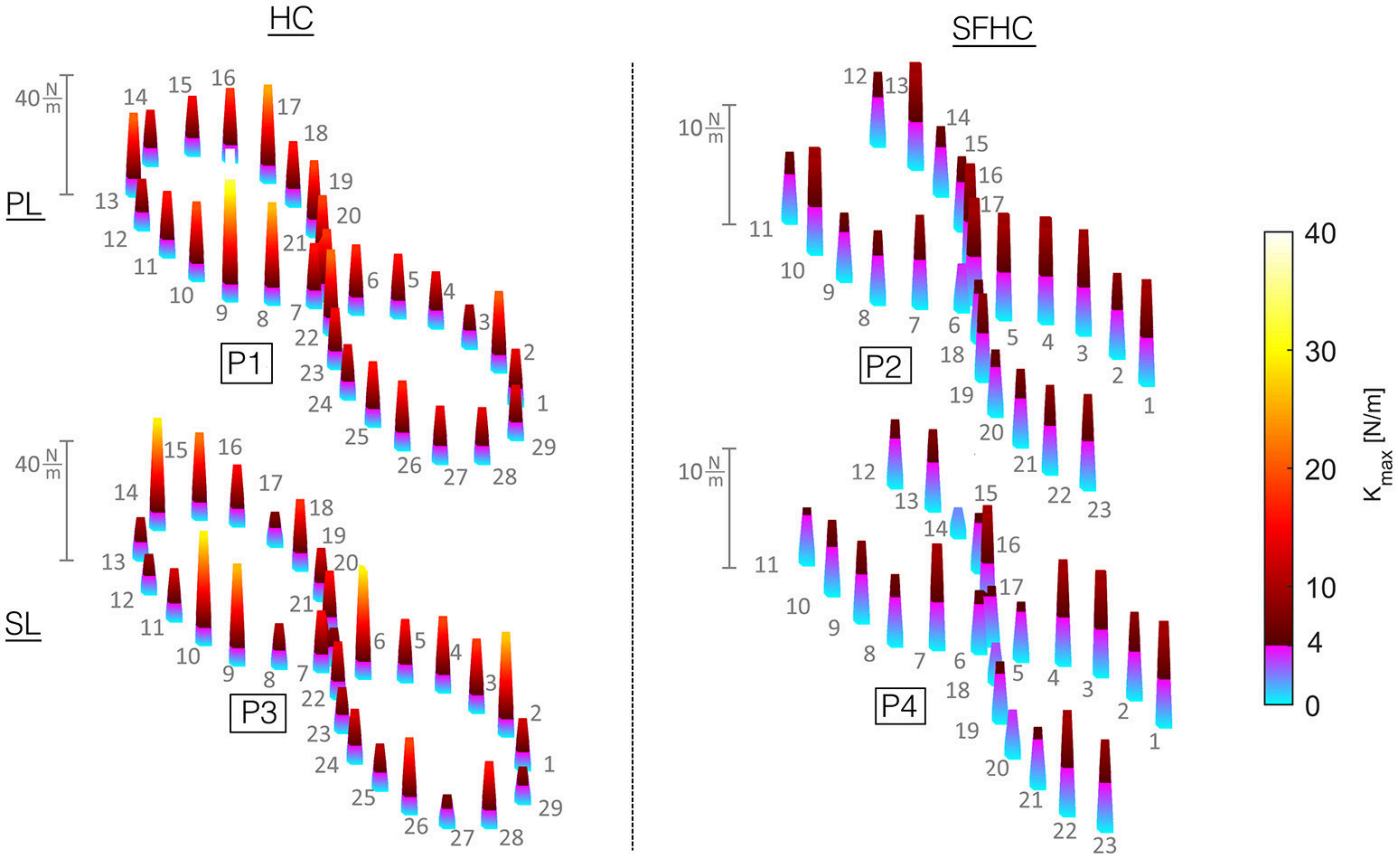
Maximal stiffness *K*_*max*_ from all the users and trials in each point along the trajectory is presented: the height and color of each column indicates the corresponding point median. To account for the difference in terms of median values from experiments PL1 and SL1 compared with PL2 and SL2, a custom color-map was designed. *K*_*max*_ values from 0 to 4 N/m are plotted with colors from light blue to purple, while *K*_*max*_ values from 4 to 40 N/m range from a dark red to bright yellow.

To further analyze stiffness modulation through the 29 (for HC) and 23 (for SFHC) points along the trajectory while teleoperating with the PL and SL master devices, Figure 6 shows the median values obtained for each experiment. The results are presented as a tri-dimensional graph where each point mean *K*_*max*_ value is represented as a colored column (see Figure 6). No evident trends can be found in stiffness maximal values modulation through the trajectory points.

**Figure 7:**
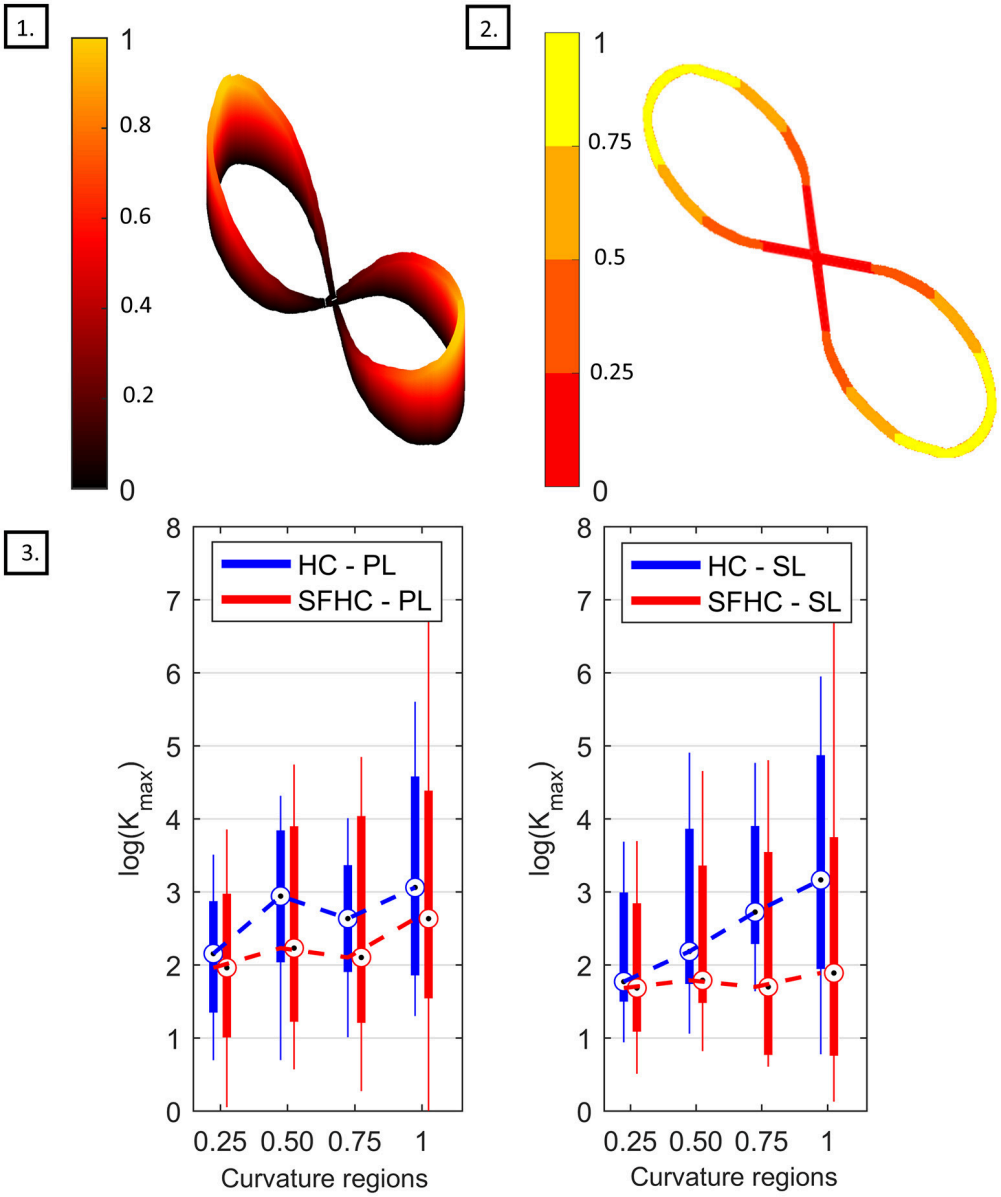
Stiffness with respect to curvature analysis. (1) Normalized curvature through the trajectory. (2) Definition of the four regions of normalized curvature. (3) Box-plot comparing the maximal stiffness value *K*_*max*_ for the PL master device, on the left, and for the SL master device on the right. The boxes represent the first and third quartile, while the whiskers represent the minimal and maximal values.

Regarding the end-point stiffness mean values relation with curvature, the three-way ANOVA showed significant difference [*F*_(3, 624)_ = 28.19, *p* < 0.0001] between the four different levels of curvature. The box-plots in Figure 7, 3 show the maximal stiffness distribution as function of four levels of trajectory curvature for PL and for SL. Although characterized by high interquartile distances, the similar behavior can be observed for PL and SL and in the two tasks: the smallest *K*_*max*_ mean values are registered where the normalized curvature is <0.25 while the maximal values appear in the range between 0.75 and 1. Interestingly, while for the hybrid parallel-serial link master device (PL) the end-point stiffness behavior in HC and SFCH shows the same trend, with an apparent plateau in the middle curvature zones, different behaviors can be seen with SL. In this case, HC shows a clear increase in the mean value of maximal stiffness with increasing levels of curvature, while in SFHC users were eliciting very small variations of arm end-point stiffness. Due to the high variance in the data, Pearson correlation analysis showed non-significant (*p* > 0.05) correlations for all the experiments. Furthermore, regarding the stiffness variability in the four regions of curvature, the three-way ANOVA test showed no significant differences [*F*_(3, 624)_ = 1.54, *p* = 0.20].

### 3.2. Stiffness against hand speed and acceleration

**Figure 8:**
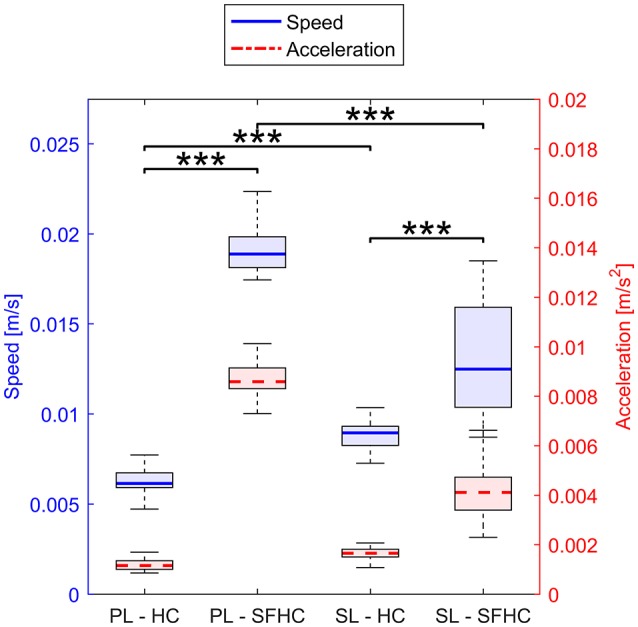
Distribution of speed (in blue, solid line) and acceleration (in red, dashed line) in the four experiments. Horizontal lines over the boxes indicate statistical difference while the number of stars indicate different levels of significance (^***^p < 0.0001).

**Figure 9:**
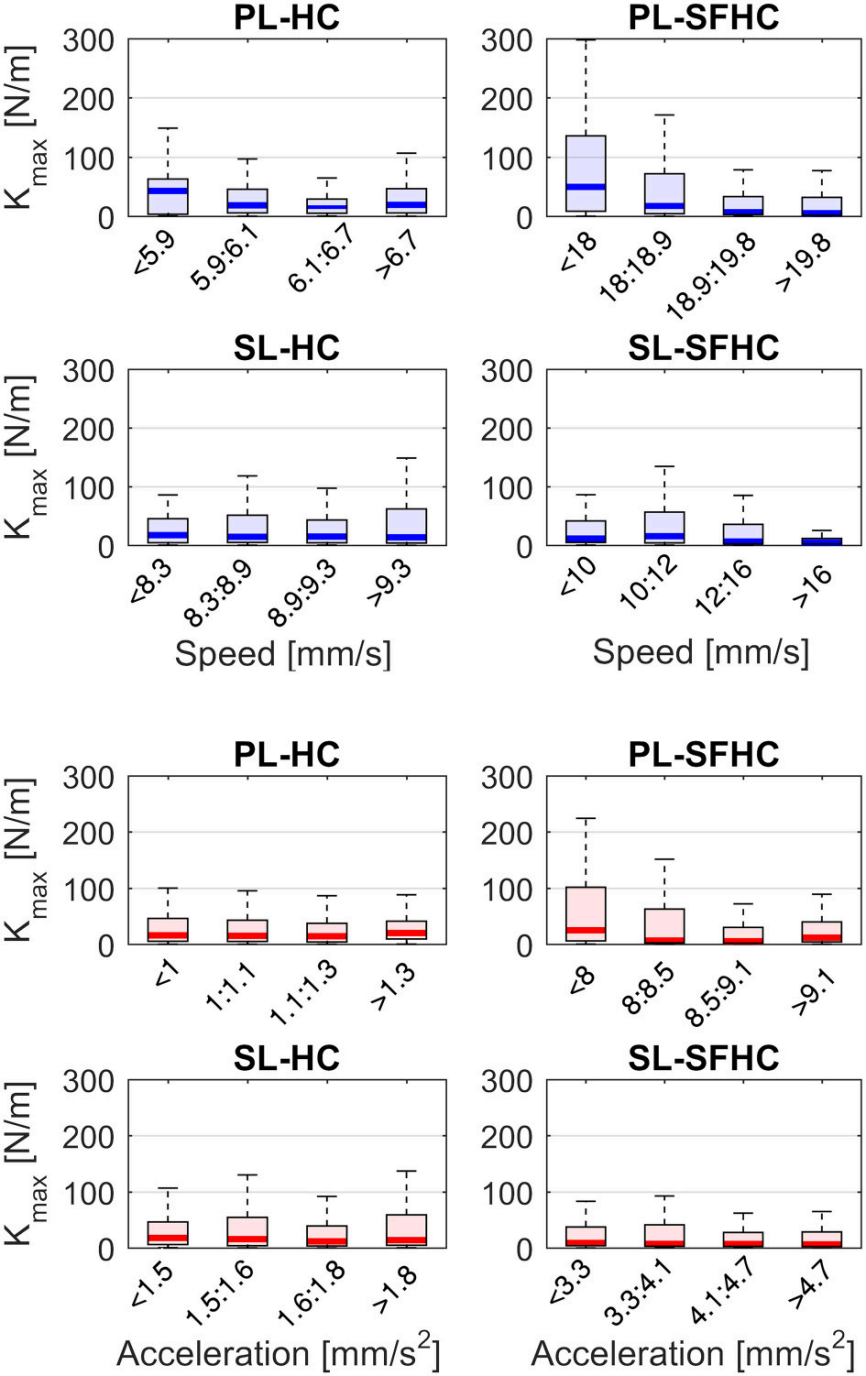
End-point stiffness distribution with respect to four increasing levels of hand speed (in blue) and acceleration (red). The levels of speed and acceleration correspond to each experiment distribution quartiles.

Figure 8 shows the speed and acceleration distribution in the four experiments; the two separated two-ways ANOVAs showed significant differences in tasks and master devices in both speed [*F*_(1, 156)_ = 998 *p* < 0.0001 and *F*_(1, 156)_ = 36 *p* < 0.0001, respectively] and acceleration [*F*_(1, 156)_ = 1,300 *p* < 0.0001, *F*_(1, 624)_ = 180 *p* < 0.0001, respectively]. The two separate three-way ANOVAs showed no significant interaction among task, master device type and level of speed or acceleration; a main significant effect was found for the task type [*F*_(1, 624)_ = 10.23, *p* < 0.005 when the data was grouped in levels of speed and *F*_(1, 624)_ = 10.23, *p* < 0.005 for levels of acceleration] while no significant effects were found for the master device type and the levels of speed or acceleration. Figure 9 shows the end-point stiffness distribution with respect to increasing levels of speed and acceleration for the four experiments. To emphasize the differences in the stiffness distribution, the non-normalized stiffness values are presented. The Pearson correlation tests between the log-normalized data and the hand speed and acceleration in the four experiments showed low (|*p*| ranging from 0.1 to 0.3) non significant (ρ > 0.1) negative correlations.

### 3.3. Distance metric

The mean distance value for each repetition was obtained for each of the 10 repetitions and users and the obtained data sets were grouped in the four experiments. The results of the two-way ANOVA with subjects' number as random-effect parameter showed a difference between HC and SFHC [*F*_(1, 156)_ = 548 *p* < 0.0001] and no difference between PL and SL [*F*_(1, 156)_ = 2.43 *p* = 0.1214]. The users were capable of achieving the best performances, testified by lower distances, with the parallel link master device (PL) and in the first task (HC). Similarly as seen with the maximal stiffness values (see Figure 4), the interquartile distances for SFHC are significantly higher than in HC.

## 4. Discussion

We evaluated the arm end-point stiffness modulation adopted by novice teleoperators in performing two tasks with two different master devices as a reflection of the control strategies adopted by the central nervous system to increase the hand resistance to internal and external noise. Using kinematic and muscular parameters, we estimated the stiffness and its relation with the trajectory characteristics and the hand speed and acceleration.

**Figure 10:**
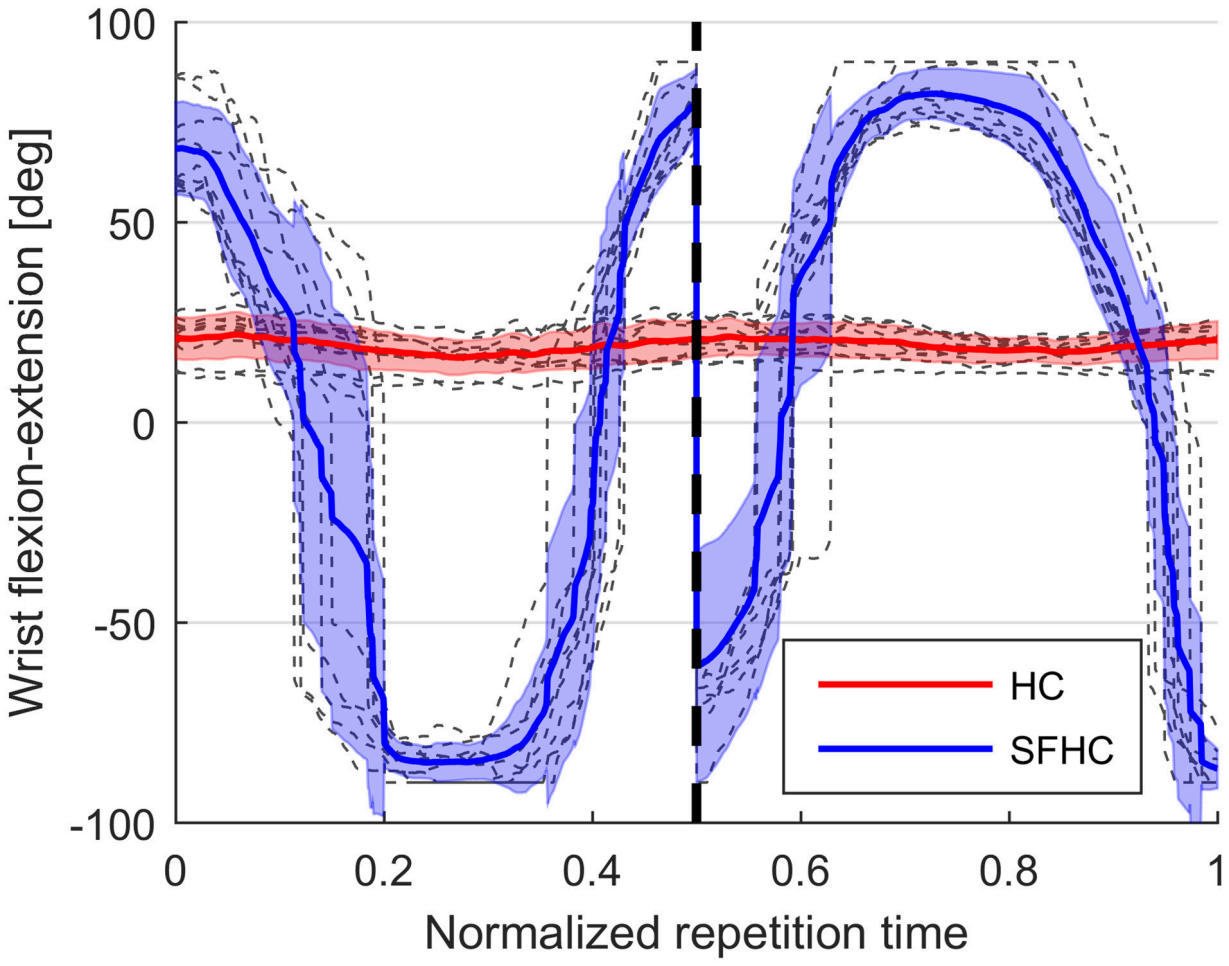
Wrist flexion-extension patterns during the execution of HC and SFHC for a single user: in gray the single normalized task repetitions, red and blue lines represent the mean joint angle signal for HC and SFHC, while red and blue areas represent the standard deviation interval.

The difference in maximal stiffness values between the different task types (as presented in Figure 4) proves that the users were adapting their stiffness modulation strategies to the different task characteristics. We were expecting higher values of end-point stiffness during the execution of the SFHC task, due to the increased complexity added by the requests to orient the tool; instead, the stiffness is significantly higher in the HC tasks. A possible explanation to this result may be that the users were discouraged to increase the arm stiffness in order to comfortably activate the wrist joint. In fact, to increase the overall arm stiffness, it would be necessary to increase the level of co-contraction also for the wrist flexor-extensors, potentially impairing the free rotation of the wrist. Therefore, this significant difference in stiffness values between HC and SFHC may be explained by the different levels of wrist flexion-extension activations that were registered in the two tasks. As an example, Figure 10 shows the wrist flexion-extension patterns found during the execution of HC and SFHC for a single user with the PL master device. The same significant difference can be seen in all the acquired users.

The fact that users were eliciting similar values of stiffness when teleoperating with the two master devices seems to suggest that users can compensate for the significant differences in the master devices mechanical proprieties assuring the same overall dynamic performances.

Users showed the highest end-point stiffness variability when teleoperating with the serial link robot during the execution of the HC task, possibly hinting that for that specific task, the SL master device's characteristics offered a less stable and reliable interaction with respect to PL. Interestingly, while for the PL master device the request to orient the tool along the trajectory introduced higher variability in the elicited stiffness, the opposite can be seen for SL. This discrepancy could be caused by the significant differences in the master device structural construction: in PL the hand rotations are completely decoupled from translations while in SL rotations are less hand-centered. This finding would therefore endorse the hypothesis that different kind of tasks may require different master device mechanical characteristics.

The statistical difference in arm stiffness mean values between different regions of curvature could suggest a relation between trajectory curvature and stiffness maximal values: in fact, the users were generally generating the lowest stiffness in the low-curvature portion of the trajectory. Under the hypothesis that straight trajectories are easier to follow than highly curved ones, it is possible that the users were relying on higher stiffness to increase their performance in the most difficult parts of the trajectory. On the other hand, this modulation seems particularly affected by the task and master device characteristics. By looking at the stiffness distributions in Figure 7, it seems that while the PL master device offers a rather consistent interface for both tasks, allowing more or less the same modulation in HC and SFHC, the SL master device showed different behaviors in the two tasks. Specifically, while an increase in the mean stiffness at high curvature can be seen for the HC task, arm end-point stiffness was almost constant for SFHC. This discrepancy may suggest that, during the SFHC task performance, when teleoperating with the SL master device, users weren't able or didn't felt the necessity to adopt the same kinetic strategy that they used otherwise.

The disparity in the speed and acceleration distributions between the two tasks could lie in the differences in the task graphical representation: in order to make the SFHC task more clear, the trajectory thickness is increased with respect to the HC. In the same way, the tool-tip changes from a small point (in HC) to a cylinder (in SFHC). These differences could have decreased the users perception of the distance from the target trajectory thus reducing the feedback on their actual performance. Consequently the users performed the task with higher speed. This explanation seems to be endorsed by the statistically higher mean distances recorded in SFHC with both master devices. The differences in mean speed and acceleration between the master devices are affected by the task request: during HC users where slower using PL while significantly faster using SL. On the contrary, the maximal speed for SFHC was obtained with PL, showing how different tasks may require different types of master devices in order to be efficiently executed. No statistical difference was found between the arm stiffness at different levels of speed and acceleration, but the higher end-point stiffness values and variability that were seen for some experiments, suggest that, in those experiments, high stiffness is more likely to occur at lower speed and accelerations. On the other hand, it appears that, even though joint speed and acceleration are fundamental components in the joint stiffness computation, their relation with arm end-point stiffness can't be modeled with simple correlations.

A limitation of this work can be found, first of all, in the small number of subjects; the number of trials for each task may not have been enough to account for the high variability that characterizes human motor control. Another limitation lies in the described differences between the two tasks; the SFHC task, in fact, differs from HC not only for the necessity or re-orienting the tool end-effector, but also in terms of trajectory thickness and tool-tip dimension.

In conclusion, the results obtained suggest that the users tend to modulate the arm endpoint stiffness with respect to different tasks and interfaces and that this modulation is influenced by both the trajectory characteristics and the users' hand kinematics. The users were coping with the difference in task design and master device by adapting their arm stiffness modulation both in terms of central tendencies and variability. Although affected by some limitations, these findings prove that the arm dynamic proprieties are highly variable and that there could be significant benefits from the estimation of the kinetic proprieties of the users' arm during teleoperation. For instance, knowing how the users modulate their stiffness would allow to develop master devices able to match and to enhance this modulation, potentially reducing the energetic cost of the teleoperators while maintaining high precision. The results obtained could also be used to improve human-robot interactions during cooperative tasks, as in Beretta et al. (2015a,b).

### 4.1. Future developments

An interesting possible future development would be the inclusion in the research of a higher number of subjects as well as expert teleoperators, in order to study and compare the stiffness modulation strategies that they may have developed with expertise. To further understand which are the characteristics and parameters that play a fundamental role in the human-robot interaction, it would also be advisable to study how stiffness relates to other dynamic parameters such as damping and inertia whose relation to human-robot system stability have been recently investigated (Dyck et al., 2013). These studies proved that the human arm behavior can change from passive to active based on the task performed and on the magnitude of the force perturbation applied.

## Author contributions

JB developed the acquisition framework, system synchronization software, performed the experiments, analyzed data and wrote the paper; with ED, he conceived the methods and analysis. JJ helped in designing and performing the experiments, also writing the corresponding portion of the manuscript. GF and ED supervised, reviewed and approved the work.

### Conflict of interest statement

The authors declare that the research was conducted in the absence of any commercial or financial relationships that could be construed as a potential conflict of interest.
